# Lost diagnoses in not otherwise specified headache in Emergency Department

**DOI:** 10.1007/s13760-021-01687-1

**Published:** 2021-08-27

**Authors:** Antonio Granato, Laura D’Acunto, Maria Elisa Morelli, Giulia Bellavita, Franco Cominotto, Paolo Manganotti

**Affiliations:** 1 Clinical Unit of Neurology, Headache Centre, Department of Medicine, Surgery and Health Sciences, University Hospital and Health Services of Trieste—ASUGI, University of Trieste, Strada di Fiume 447, 34149 Trieste, Italy; 2 Emergency Department, University Hospital and Health Services of Trieste, Strada di Fiume 447, 34149 Trieste, Italy

**Keywords:** Headache, Not otherwise specified headache, Emergency Department headache, Consulting visits

## Abstract

The diagnosis of Not Otherwise Specified (NOS) headaches in the Emergency Department (ED) is frequent despite many specialist visits performed. The aim of the study was to examine specialist visits carried out in the patients discharged from ED with diagnosis of NOS headache to evaluate discrepancies between specialist and ED diagnosis at discharge. We retrospectively (1.6.2018–31.12.2018) analyzed all the patients admitted with non-traumatic headache to the ED of the tertiary-care University Hospital of Trieste. We evaluated the patients discharged from ED with a final diagnosis of NOS headache and who underwent at least one specialist examination. Demographic data, specialist and ED diagnosis were analyzed.  One hundred twenty-four patients (93 F, 31 M, mean age 44 ± 15 years) were included. 71.8% of patients were examined only by a neurologist, 12.9% by non-neurologists, 15.3% by both neurologist and non-neurologist. Only 37% of the patients received a precise diagnosis. Neurologist made a diagnosis slightly more frequently than the other consultants (40.5% vs 37.5%). Neurologists diagnosed primary headaches, headaches secondary to neurological diseases, and facial neuralgia, instead non-neurologists diagnosed only headaches secondary to non-neurological diseases. Primary headaches were diagnosed in 25.7% of cases, migraine being the most frequent. Physicians did not report any specialist diagnoses in the ED discharge sheet. Specialist consultants made specific diagnoses in about one-third of patients that were not reported as final in the discharge records by the ED physician. This leads to a loss of diagnoses and to an overestimation of NOS headache.

## Introduction

Non-traumatic headache (NTH) accounts for 0.4–5% of all Emergency Department (ED) visits, and for 13–27.8% of all neurological visits in the ED [1–3]. The diagnostic framework of headache in the ED constitutes a challenge for Emergency Physicians (EPs) and specialist consultants. The prevalence of primary headaches ranges between 1.2 and 2.35% of all visits in the Italian EDs [4, 5]. However, secondary headaches stemming from serious underlying pathologies comprises 4–5% of all ED attendances [6–8]. Despite the introduction of ICHD-3 criteria and of red and green flags differentiating between primary and secondary headache [2, 9], not otherwise specified (NOS) headache still remains a frequent ED diagnosis. The role of specialist consultants to improve diagnostic accuracy of headache in ED is being debated.

The aim of this study was to examine specialist visits carried out in all the patients discharged from ED with diagnosis of NOS headache and to evaluate discrepancies between specialist diagnoses and ED diagnosis at discharge. Secondly, we analyzed the reasons why the EPs do not use the correct diagnosis of headache and the management and therapeutic recommendations provided at ED discharge.

## Patients and methods

We retrospectively analyzed all the patients admitted to the ED of the tertiary-care University Hospital of Trieste (Italy) because of non-traumatic headache during a 6-month period (from 1.6.2018 to 31.12.2018). We took into account the patients who were discharged to home from our ED with a final diagnosis of NOS headache and who underwent at least one specialist examination in our ED.

The presence of specialist consultants, including neurologists, is available 24 h/day.

To analyze the most common reasons why the EPs do not use the correct diagnosis of headache in ED, two neurologists experienced in headaches (AG and LDA) structured an anonymized questionnaire assessing the knowledge and the application of ICHD-3 criteria in ED setting in relation to the presence of specialist consultants in ED and the EPs friendliness with medical software. The questionnaire included five questions, and the EDs could answer with multiple choices (Table 1). The questionnaire was delivered by hand to each emergency physician.

**Table 1:** Questionnaire for Emergency Physicians about knowledge of ICHD-3 criteria and application of ICHD-3 criteria in emergency setting

I don't know the ICHD-3 criteria
I use the ICHD-3 criteria for the diagnosis of headache
I know the ICHD-3 criteria but they are too complex to use in the emergency setting
I know the ICHD-3 criteria but I prefer to contact the specialist consultant (neurologist or other)
I know the ICHD-3 criteria but It is difficult or too long to find the precise diagnosis on PC medical software

*ICHD-3* The International Classification of Headache Disorders 3rd edition

For each patient, we examined demographic and clinical data, diagnostic procedures, specialist examinations, discrepancies between specialists and ED diagnosis at discharge. We also analyzed the presence at discharge of recommendations of the specialist consultant, of indications to refer to the Headache Centre, and therapeutic prescriptions for headache. All the data were collected using the digital hospital medical records.

Subsequently, the data were entered in an ad hoc database and analyzed with SPSS Statistics 22.0. Each patient provided a written and signed informed consent allowing the analysis of his/her data for clinical and research purposes. The study was conducted according to the principles of the Declaration of Helsinki.

## Results

From 01.06.2018 to 31.12.2018, 463 (1.2%) out of 38.582 patients were admitted to the ED because of NTH. One hundred twenty-four (26.8%) of them fitted the inclusion criteria and were analyzed. The mean age of patients was 44 years (SD 15 years, range 17–87), with a prevalence of women (75%).

Out of all EPs (*n* = 30), 25 (84%) completed the questionnaire evaluating the application of ICHD-3 criteria in Emergency Room. Twenty four EPs (96%) did not know ICHD-3 diagnostic criteria, while only one physician answered that he knew the ICHD-3 criteria but they were too complex to use in the emergency setting.

One hundred and eight subjects underwent a neurological examination (87.1%), while non-neurologist consultants visited 35 patients (28.2%). Eighty-nine patients (71.8%) were evaluated only by a neurologist, 16 (12.9%) by other non-neurologist consultants, 19 (15.3%) received both neurological and non-neurological examinations (Table 2).

**Table 2:** Specialist consultants

Specialist consultants	Patients [*n* (%)]
Neurologist Neurologist + non-neurologist consultants Otolaryngologist Oculist Specialist in infectious disease Orthopedist Odontostomatologist Non-neurologist consultants Otolaryngologist Oculist Specialist in infectious disease	89 (71.8%) 19 (15.3%) 7 (5.6%) 5 (4.1%) 3 (2.4%) 2 (1.6 %) 2 (1.6%) 16 (12.9%) 8 (6.5%) 6 (4.8%) 2 (1.6%)

A cranial CT scan was performed in 50.1% of subjects. About half of the patients evaluated by the neurologist (52.8%) underwent a cranial CT scan. A lumbar puncture was performed in 2.4% of patients.

A definite diagnosis was made only in 46 (37%) patients, with primary headaches being the most frequently identified disorder (25.7%).

In 40.5% of all neurological examinations, the neurologist made a precise diagnosis (Table 3). The neurologist diagnosed all primary headaches, as well as the secondary headaches related to neurological diseases (transient ischemic attack, alcohol-induced headache, sleep apnea headache, hypoglycemia), and all the neuropathies and facial pains. Non-neurologists made a precise diagnosis in 37.5% of their visits, and all the diagnoses confirmed the presence of headaches secondary to non-neurological diseases. Among the 19 patients subjected to both neurological and non-neurological consultations, four had specific diagnoses: the neurologist diagnosed the two patients discharged with diagnosis of primary headache, while the non-neurologist consultant diagnosed the two patients discharged with diagnosis of secondary headache (Table 3).

Among the primary headaches, migraine was the most frequent diagnosis (20.9%), while tension-type headache represented a small part of the whole sample (4%). Among the diagnoses of secondary headaches, only one patient had a headache secondary to a life-threatening disease (transient ischemic attack), whereas all the other diagnoses were related to benign pathologies (Table 3).

Seventy-two patients (58.1%), including all 46 patients with a definite specialist diagnosis and 26 out of the 78 patients with NOS headache, received therapeutic prescription at ED discharge. All the patients who did not receive any therapy at discharge were referred to the Headache Centre. Symptomatic medication was prescribed in 62 patients (51%), the most frequent being NSAIDs (78.7%). Triptans were recommended in only 8.2% of the cases, despite the high percentage of patients suffering from migraine. A prophylaxis, alone or in association with symptomatic drug, was indicated in ten patients (Table 4).

**Table 3:** Diagnoses of specialist consultants

Diagnosis	Consulting visits			Total visits (*n* = 124)
	Neurological (*n* = 89)	Non-neurological (*n* = 16)	Neurological + Non-neurological (*n* = 19)	
Primary Headache Migraine without aura Migraine with aura Tension-type headache Cluster Headache	30 (33.7%) 20 (18.5%) 5 (5.6%) 4 (4.5%) 1 (1.1%)	– – – – –	2 (10.5%) 1 (5.3%) – 1 (5.3%) –	32 (25.7%) 21 (16.9%) 5 (4.0%) 5 (4.0%) 1 (0.8%)
Secondary Headache Transient ischemic attack Headache attributed to systemic viral infection Alcohol-induced headache Sleep apnoea headache Hypoglycaemia Headache attributed to disorder of the eyes Headache attributed to disorder of ears Headache attributed to acute rhinosinusitis Headache attributed to disorder of the teeth	4 (4.4%) 1 (1.1%) – 1 (1.1%) 1 (1.1%) 1 (1.1%) – – – –	6 (37.5%) – 1 (6.2%) – – – 3 (18.8%) 1 (6.2%) –) 1(6.2%)	2 (10.5%) – 1 (5.3%) – – – – – 1 (5.3%) –	12 (9.7%) 1 (0.8%) 2 (1.6%) 1 (0.8%) 1 (0.8%) 1 (0.8%) 3 (2.5%) 1 (0.8%) 1 (0.8%) 1 (0.8%)
Neuropathies and facial pains Trigeminal neuralgia Occipital neuralgia	2 (2.2%) 1 (1.1%) 1 (1.1%)	–	–	2 (1.6%) 1 (0.8%) 1 (0.8%)
None specific diagnosis	53 (59.5%)	10 (62.5%)	15 (79.0%)	78 (63.0%)

**Table 4:** Therapeutic recommendations at discharge

Medications	Patients [*n* (%)]
No therapies	52 (41.9%)
Attack medications	61 (49.3%)
NSAIDs	48 (38.7%)
NSAIDs alone	42 (33.9)
NSAIDs + BDZ + antiemetics	2 (1.6%)
NSAIDs + BDZ	3 (2.4%)
NSAIDs + opioids	1 (0.8%)
Triptans	10 (8.2%)
Opioids	3 (2.4%)
Prophylaxis	7 (5.6%)
Antidepressive	3 (2.4%)
Antiepileptics	2 (1.6%)
Calcium antagonist	1 (0.8%)
Muscle relaxers	1 (0.8%)
Prophylaxis and attack	4 (3.2%)
Antidepressive + BDZ	1 (0.8%)
Antidepressive + NSAIDs	2 (1.6%)
Muscle relaxer + NSAIDs	1 (0.8%)

*NSAIDs* nonsteroidal anti-inflammatory drugs; *BDZ* benzodiazepines

No diagnosis made by the specialist consultants was reported as final in the ED discharge reports. However, the EPs suggested following the recommendations made by the specialist consultants in 76.6% of the discharge reports.

Other diagnostic tests were indicated at discharge in 20.9% of patients, including MRI brain (12 patients, 9.6%), EEG (one patients, 0.8%), blood test including thrombophilia set, thyroids hormones, glycaemia (five patients, 4%), and other specialist consultations (eight patients, 6.5%). The neurological consultants requested all the diagnostic procedures (tests) recommended only in those patients who did not receive a precise diagnosis.

Ninety-five cases (76.6%) were referred to the Headache Centre: 83 patients were referred to it by the neurologist, 12 by the EPs.

## Discussion

This survey focused on the specialist visits performed in ED in all the patients discharged from ED with diagnosis of NOS headache. The aim was to evaluate the discrepancy between specialists and ED diagnoses at discharge, ED management and therapeutic recommendations provided at discharge from ED, and the reasons why the EPs do not use the correct diagnosis of headache analyzing the Emergency Physicians’ knowledge and application of ICHD-3 diagnostic criteria in the ED setting.

In our study, 124 patients who received at least one specialist visit fulfilled the criteria for NOS headache at ED discharge, most patients (87.1%) received neurological examination at least, 12.9% underwent only other specialist consultation. As previously described, consultant neurologists are often referred to for almost all cases of de novo or non-traumatic headaches [4], since they can provide an additional interpretation of neuroimaging scans. Other specialists are usually requested in presence of associated symptoms, such as vertigo, blurred vision, diplopia or fever that could be suggestive of a secondary origin.

The main result of this study is that a precise diagnosis of headache was given in only 37% of cases, but these diagnoses were not reported in the discharge reports by EPs. Neurologists made a precise diagnosis slightly more frequently than the other consultants (40.5% neurologist vs 37.5% non-neurologist examinations). Primary headaches, in particular migraine without aura, were the most frequently diagnosed. Non-neurologist specialists identified only secondary causes of headache, which were benign secondary headaches pertaining to the specific clinical area for which the specialist was being consulted. The secondary headaches identified by the neurologist were mostly secondary to non-neurological diseases (Table 3).

The problem of under-diagnosis or misdiagnosis of primary and secondary headache in ED was largely investigated from the perspective of neurology and emergency medicine, and several flow-charts were made to improve the diagnostic accuracy, especially regarding potentially dangerous secondary headache [10–12]. The role of EPs is crucial in the first assessment of a headache complaint and has two major responsibilities: to treat painful headache and to discover its underlying causes. However, a diagnostic precision in diagnosing headache is not always simple, which could lead to erroneous conclusions and may distract EPs from other important priorities [13]. Additionally, EPs may not feel confident when performing neurological or another specialist examination [14].

In our survey, we analyzed the reasons why the EPs do not use the correct diagnosis of headache, including knowledge of ICHD-3 criteria, application of ICHD-3 criteria in emergency setting, preference to contact the specialist consultant and application of ICHD-3 criteria on PC medical software. The questionnaire was completed by a high percentage of respondents. Surprisingly, almost all EPs (96%) admitted they did not know ICHD-3 criteria, suggesting this is the cause of the high rate of NOS headache diagnoses in ED and why EPs tend to refer patients to specialist consultants.

Previous studies analyzed neurological examinations in ED focusing on motivations and frequencies of consultation, describing the diagnostic accuracy and the concordance rate between consultants and ED physicians [15–17]. *Moeller *et al*.* *2008* reported that misdiagnosis or diagnostic uncertainly occurred in one-third of all neurological consultations in ED, especially for benign neurological conditions such as migraine. They underlined a 35.7% discordance between the initial diagnosis of EPs and a neurological diagnosis. *Hansen *et al*.* *2015* instead found a 85.1% concordance between the ED physician and the consultant neurologist. Finally, *Sahai-Srivastava *et al*.2008* indicated that 79% of final diagnosis in ED matched the diagnosis of neurologist [18], while in an Italian study a lower agreement was found for cases admitted to EDs with a first attack (de novo headache) [19].

NOS headache represents the most frequent diagnosis of discharge from ED among non-traumatic headache [8, 18, 20, 21]. In our sample, we found that 37% of patients received a precise diagnosis which was not reported in the ED discharge reports. This leads to an overestimation of NOS headache discharge diagnosis. Our data suggest that NOS headaches consist predominantly of primary headaches, mostly migraine without aura. Our data are in line with a study of *Viganò *et al*.* who re-evaluated in a Headache Unit a group of patients who had previously been referred to the ED because of headache. ED diagnosis at discharge was NOS headache in 65.3% of cases. All the patients were reclassified in the Headache Unit as primary headache (96.8%) and secondary nonlife-threatening headache (3.2%). The majority of patients (56.2%) were reclassified as migraine without aura [21]. In another recent study, a re-classification of diagnoses of all patients discharged by ED in 2.5 years was made according to the ICHD-3 criteria. This re-classification found an overestimation of tension type headache and confirmed migraine as a common and underdiagnosed headache [22].

In our series, consultants in ED did not give a precise diagnosis of headache in 63% of patients. There are different possible reasons for this high percentage, for example anamnestic and clinical data could not be sufficient to implement the ICHD-3 criteria, or consultants could be put under pressure by other emergency consultations in ED, so sometimes it is not possible to give an exhaustive conclusion.

The ICHD-3 criteria seem to be a reliable help to distinguish primary from non-primary headache, in particular if associated to red flags [2]. However, these criteria are not well known and are not always easy to apply in the Emergency Room [23, 24].

All the patients who received a specific diagnosis by consultants and only 35.9% of patients without diagnosis received therapeutic prescriptions at discharge. The high frequency of patients not receiving treatment at discharge may depend on the absence of a specific diagnosis and on the fact that all these patients were referred to the Headache Centre by EPs, where specific diagnoses and prompt therapies would be given. NSAIDs remain the most prescribed drugs on discharge, while triptans and similar prophylaxis were recommended only in few cases, in line with other studies [6, 25].

Some limitations of the study include the retrospective design, missing data about anamnestic information, acute therapies administered in ED and clinical outcome. Authors conducted no direct interviews with patients or emergency physicians.

In conclusion, specialist consultants made a specific diagnosis only in about one-third of the patients, which was never reported as final diagnosis in the discharge records by the ED physician. This may depend on the fact that, in busy moments, once the specialist consultants have confirmed the diagnosis of primary or benign secondary headache, EPs could just confirm the diagnosis of admission to accelerate the patient’s discharge, and suggested to read the consultant's report. This fact leads to a loss of diagnoses specifically made by specialists in ED and to an overestimation of NOS headache discharge diagnoses. Neurologists in particular made diagnoses only in 40.5% of all their visits, and primary headaches were the most frequent ones. These data underline the risk to underestimate primary headaches in the ED. All the patients who had specific diagnoses received therapeutic prescription at discharge from the ED. Although there were many patients with primary headache, specific treatments such as triptans were rarely prescribed. Many patients have been referred to the Headache Center. Referring patients to the Headache Center may relieve the ED physician and consultants from making diagnosis and giving specific treatment. Finally, the EPs lack of knowledge of ICHD-3 criteria may partially explain the high rate of NOS headache diagnoses in ED. An ICHD-3 diagnostic criteria educational program tailored to the ED setting could increase the use of diagnostic criteria with reduction of unspecified headaches.

## Abbreviations

BDZ: Benzodiazepines
ED: Emergency Department
EPs: Emergency Physicians
NSAIDs: NSAIDs non-steroidal anti-inflammatory drugs
NTH: Non-traumatic headache
NOS: Not otherwise specified
